# The Dynamic Tunability of Memristor-Based Active Filters

**DOI:** 10.3390/mi14112064

**Published:** 2023-11-05

**Authors:** Ivo Marković, Milka Potrebić Ivaniš, Dejan Tošić

**Affiliations:** 1 Earth & Environmental Sciences Area, Lawrence Berkeley National Laboratory, Berkeley, CA 94720, USA; ivomarkovic@lbl.gov; 2 School of Electrical Engineering, University of Belgrade, 110000 Belgrade, Serbia; tosic@etf.rs

**Keywords:** active filter, memristor, memristor programming, tunable filter

## Abstract

When the memristor was fabricated for the first time, it launched an entirely new field of research. Many of the published papers regarding memristors are primarily theoretical and are based on computer simulations. Some recent papers analyze the memristor’s programming circuits, but to the best of the authors’ knowledge, no memristor has been embedded into a commercial analog circuit. This paper is practically oriented and it is based on the experimental results obtained by measurements on the circuit prototype. We present a solution for automated programming of a commercially available memristor and its implementation in tunable active bandpass filter design. The novelty of this paper is that the active bandpass filter’s central frequency could be programmed during the filter operation, so a pause for memristor state-switching is not required. The experimental results are promising, and open up possibilities for the memristor’s application in analog systems.

## 1. Introduction

Memristors have garnered significant interest since their initial fabrication [1]. Numerous theoretical papers have explored potential memristive applications across various fields. Some applications in analog and digital electronics include a memristor-based oscillator for weak signal detection [2], a third-order Wien bridge oscillator [3], and a programmable threshold comparator [4]. Memristor modeling for a common-source amplifier was presented in [5]. In [6], the authors propose an FPGA IP core for both analog and digital applications. Memristor fabrication for RF/microwave electronics is documented in [7].

In RF/microwave circuit design involving memristors, different authors have proposed electromagnetic devices [8], power dividers, coupled resonator bandpass filters [9], reconfigurable microwave filters [10,11], and phase shifters [12]. An implementation using commercially available memristors in digital electronics is also documented [13]. Recently, a group of researchers presented the results of commercially available memristor programming using the Digilent Analog Discovery 2 (AD2) instrumentation tool [14,15,16,17]. While there is research reporting practical realizations in analog electronics [18], implementing memristor programming within an inverting amplifier, a phase shifter, and a highpass filter requires operational pauses.

Memristors are promising circuit elements for numerous analog and digital filtering applications. Memristive filter design is one of the most frequently discussed topics in the contemporary literature. The primary approach in filter design is to use the memristor either as a switch for reconfigurability or as a variable resistor for adjusting the filter frequency response. One of the pioneering studies on memristive filter design was proposed by the research group led by Professor Tetzlaff [19]. This group explored passive first-order lowpass and second-order bandpass filters enhanced by memristors. The central concept is the substitution of a resistor with a memristor. The memristor allows for the fine-tuning of the filter frequency response through memristance programming. The study demonstrates how memristance influences the filter’s cutoff frequency and the quality factor of the frequency response for both lowpass and bandpass filters.

The introduction of memristive components into filter design is exemplified in a study of a lowpass filter where the authors replaced a resistor with a memristor and a capacitor with a memcapacitor [20]. A potential reconfigurable FIR filter design with memristive weights was analyzed by Professor Strukov and his research group [21]. This mixed-signal filter represents a more efficient hybrid circuit achieved by using a DAC circuit with dot-product operation.

The practical applications of TiO_2_ memristors in tunable active filters are showcased in [22]. Here, memristors function as tunable loads in filter designs like the Sallen–Key active lowpass and bandpass filters. The underlying principle is that a specified memristance value allows for the tuning of filter characteristics such as passband frequencies and quality factors. Another instance of a tunable first-order active highpass filter employing a TiO_2_ memristor is designed in [23]. Commercial KnowM memristors are recommended for RF passive filter implementations, as illustrated in [24], where the lowpass filter bandwidth can be adjusted by varying the memristance value.

In [25], the relationship between filter cutoff frequency and memristance value is explored for a second-order active lowpass filter. An active bandpass filter incorporating a parallel memristor and capacitor is introduced in [26]. This proposal examines the stability aspect contingent on the memristor’s initial condition. A CMOS-memristor-based OTA is employed for a second-order highpass filter design in [27].

For a detailed analysis of memristors, some researchers have suggested the use of memristor emulators in various applications, including filters. An overview of these emulators has been evaluated for adaptive filtering applications to better understand their strengths and weaknesses [28]. Changing memristance permits the adjustment of the filter’s cutoff frequency. Initially, in the published research, authors recommended substituting resistors in conventional analog circuits with memristor emulators to achieve superior circuit performance. To illustrate this, the authors utilized standard lowpass, highpass, and bandpass filters. Another implementation of a memristor emulator for an active lowpass filter is presented in [29], where the memristance value governs the filter’s cutoff frequency.

A couple of amplifier circuits were analyzed in [30], where the authors demonstrated that a memristor-based voltage gain amplifier (VGA) exhibits significantly lower total harmonic distortion (THD) compared to VGA topologies with MOSFETs operating in the triode regime. In the proposed amplifier design, the memristor can switch states during operation, but the amplifier cannot function during memristor state transitions. The programming signal is amplified alongside the input signal, leading to op-amp saturation. This analysis is also applicable to the topologies studied in [30]. In [31], the authors suggest using a voltage adder comprising a 4 kΩ series resistor to mitigate op-amp saturation. Unfortunately, this solution is not suitable for KnowM memristors, as a resistor of this magnitude is insufficient to prevent op-amp saturation when the memristor is in a high memristive state. To avoid op-amp saturation, a series resistor of at least 30 kΩ is required.

In [15], the authors demonstrate that a voltage of at least 0.3 V across the memristor is necessary for proper memristor programming. Consequently, with a serial resistor of at least 30 kΩ in the voltage adder, memristor programmability would be limited when in a low memristive state. In [32], the authors employ voltage levels of ±35 V in their simulations to expedite memristor switching. However, for KnowM memristors, voltage levels higher than approximately 3 V for programming are unacceptable.

This research centers on the application of a commercially available memristor produced by KnowM [33]. The primary objective was to develop a circuit that facilitates memristor programming while being embedded within a larger circuit. We propose an active bandpass filter that can be tuned by manipulating memristor resistance. We present the theoretical design and outline circuit functionality limitations. Subsequent sections showcase our experimental results, including the programming mechanism and responses in both time and frequency domains. A brief discussion is followed by the conclusion.

The significance of this research is reflected in demonstrating the advantage of using memristors over other switching components in integrated circuits. To be more specific—most other switching components require a small amount of idle time while switching from one state to another. Memristors, on the other hand, can continuously change their memristance and can be programmed without the need for any idle time.

## 2. Tunable Active RC Bandpass Filter with Memristor

**Figure 1:**
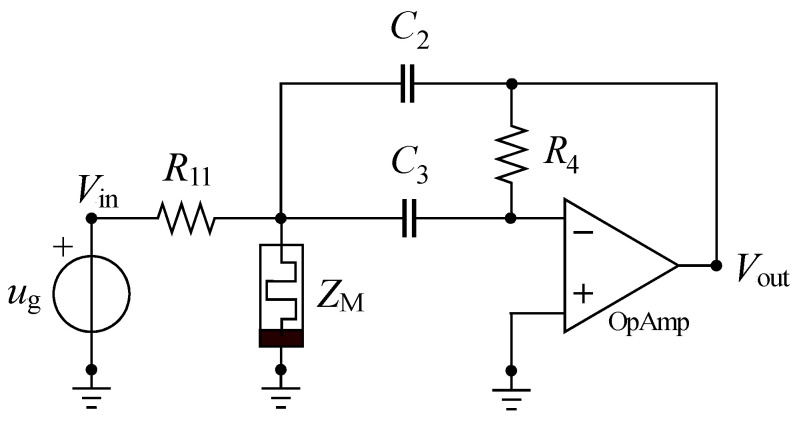
Tunable active bandpass filter with a memristor.

To eliminate the memristance programming signal from the circuit’s output, we introduced a design featuring a tunable active bandpass filter that utilizes a memristor [34]. Active filters, in general, are circuits designed to selectively amplify or attenuate input signals based on their frequency. We are specifically interested in using operational amplifiers together with RC filters to achieve bandpass characteristics, as shown in Figure 1.

The bandpass filter has a second-order transfer function as follows:

(1) $$H(s)=\frac{V_{\mathrm{out}}}{V_{\mathrm{in}}}=-K\frac{\left(\omega_{p}/Q_{p}\right)s}{s^{2}+\left(\omega_{p}/Q_{p}\right)s+\omega_{p}^{2}},$$

where $s^{2}+\left(\omega_{p}/Q_{p}\right)s+\omega_{p}^{2}=(s-s_{p1})(s-s_{p2}^{})$ and $s_{p1}$, $s_{p2}$ are a complex-conjugate pole pair, i.e., $s_{p1}=s_{p2}^{*}=s_{p}$. The parameter *K* is the gain where is assumed that K>0, $\omega_{p}$ is the central angular frequency and the magnitude of the pole $s_{p}$, and $Q_{p}$ is the quality factor of a complex-conjugate pole par.

The 3 dB bandwidth of the bandpass filter is:

(2) $$B_{3\mathrm{dB}}=\omega_{high,\;\;3\mathrm{dB}}-\omega_{low,\;\;3\mathrm{dB}},$$

where the high and low frequencies of the passband are:

(3) $$\omega_{low,\;3\mathrm{dB}}=\frac{\omega_{p}}{2Q_{p}^{}}\left(\sqrt{1+4Q_{p}^{2}}-1\right),$$

(4) $$\omega_{high,\;3\mathrm{dB}}=\frac{\omega_{p}}{2Q_{p}^{}}\left(\sqrt{1+4Q_{p}^{2}}+1\right),$$

while the 3 dB bandwidth is:

(5) $$B_{3\mathrm{dB}}=\omega_{p}/Q_{p}.$$

In the filter’s stopband, we observed a signal suppression exceeding 10 dB within the following frequency range:

(6) $$\omega \leq \frac{\omega_{p}}{2Q_{p}}\left(-3+\sqrt{9+4Q_{p}^{2}}\right)=\omega_{low,\;10\;\mathrm{dB}}$$

and

(7) $$\omega \geq \frac{\omega_{p}}{2Q_{p}}\left(3+\sqrt{9+4Q_{p}^{2}}\right)=\omega_{high,\;10\;\mathrm{dB}},$$

while for 20 dB signal suppression, the frequency range is:

(8) $$\omega \leq \frac{\omega_{p}}{\sqrt{2}Q_{p}}\sqrt{99+2Q_{p}^{2}-3\sqrt{11}\sqrt{99+4Q_{p}^{2}}}=\omega_{low,\;20\;\mathrm{dB}}$$

and

(9) $$\omega \geq \frac{\omega_{p}}{\sqrt{2}Q_{p}}\sqrt{99+2Q_{p}^{2}+3\sqrt{11}\sqrt{99+4Q_{p}^{2}}}=\omega_{high,\;20\;\mathrm{dB}}.$$

The design begins by specifying filter parameters such as the central angular frequency ω_p_, the quality factor of a complex-conjugate pole pair *Q*_p_, filter gain *K*, the capacitance *C*, and resistance ratio *X* = *R*_4_/*R*_1_, where *R*_1_ = 1/(1/*R*_11_ + 1/*Z*_M_). Refer to Figure 1 for details. Parameter $Z_{M}$ presents memristance value, which can be programmed to satisfy the filter specifications. Instead of parameter $Q_{p}$ we can specify the filter bandwidth.

The transfer function of this active *RC* bandpass filter is:

(10) $$H(s)=\frac{V_{\mathrm{out}}}{V_{\mathrm{in}}}=\frac{-s\frac{1}{R_{11}C_{2}}}{s^{2}+s\frac{C_{2}+C_{3}}{C_{2}C_{3}R_{4}}+\frac{1}{C_{2}C_{3}R_{4}}\left(\frac{1}{R_{11}}+\frac{1}{Z_{M}}\right)}.$$

When we determine the values of *C* and *X*, then we can compute the filter element values, such as:

$$C_{2}=C,$$

$$C_{3}=C\frac{X-2Q_{p}^{2}-\sqrt{X^{2}-4Q_{p}^{2}X}}{2Q_{p}^{2}},$$

$$R_{1}=1/\left(1/R_{11}+1/Z_{M}\right)=\frac{1}{Q_{p}^{}\omega_{p}(C_{2}+C_{3})},$$

$$R_{4}=XR_{1},$$

$$R_{11}=\frac{C_{2}R_{4}}{K(C_{2}+C_{3})},$$

(11) $$Z_{M}=\frac{C_{2}R_{1}R_{4}}{C_{2}R_{4}-R_{1}K(C_{2}+C_{3})}.$$

The filter is engineered for tunability, allowing for central frequency adjustments solely through modification of the memristance value (*Z*_M_), while maintaining a constant filter bandwidth.

We adopted the target central frequencies of 19.5 kHz (1st state) and 26 kHz (2nd state). We fixed the 3 dB bandwidth to the value of 3.18 kHz. The op-amp used is NE5532P (Texas Instruments, Dallas, TX, USA) [35].

**Figure 2:**
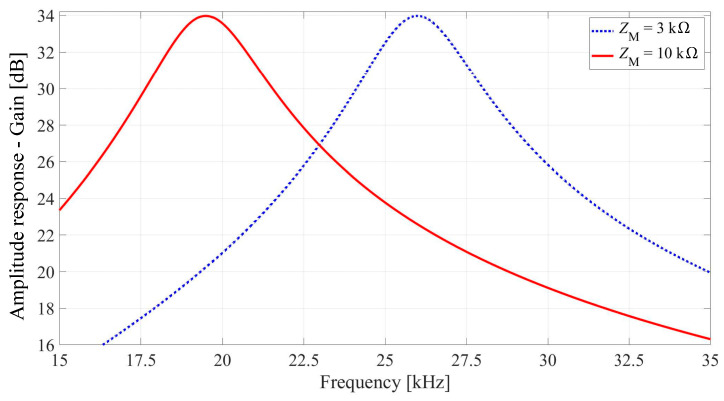
Amplitude response of the tunable active bandpass filter with a memristor.

For both states, the filter specification parameters and designed memristance values are given in Table 1, while the values of capacitors and resistors do not change, i.e., *C*_2_ = 200 pF, *C*_3_ = 200 pF, *R*_11_ = 5 kΩ, *R*_4_ = 500 kΩ. The filter amplitude response for these two cases is given in Figure 2.

## 3. Programming Signal Analysis

As stated earlier, the aim of this study is to control the memristor state, i.e., memristance value during the filter operation mode. This means that the memrsitor could change its memristance on the fly and that the control signal, i.e., the memristor programming signal, should be suppressed at the filter output. The assumption is that the control signal is added to a useful signal such as a sinusoidal signal. The objective is to propose an adjustment the control process for memristance programming, realizing an appropriate control signal for the analyzed filter. The filter must suppress that control signal. This leads to the question: what is the relationship between filter circuit elements and parameters of the control signal?

In the current literature, much research focuses on controlling the programming signal’s polarity, amplitude, and duration due to the unpredictable and challenging nature of precisely and repeatably controlling the memristor’s state [36,37,38,39,40]. Conversely, some memristance control solutions are based on various hardware implementations [36,37,38,39,40].

**Figure 3:**
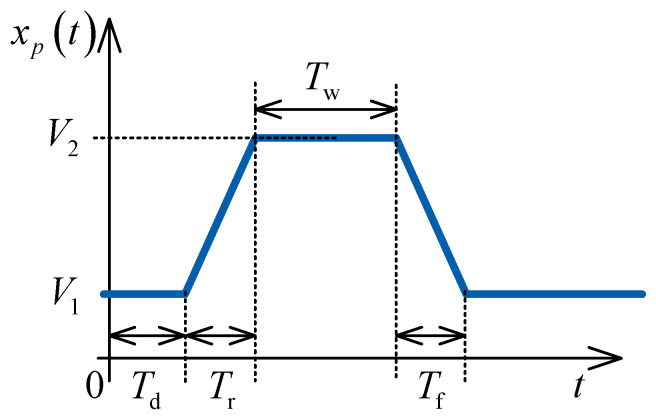
Pulse parameters.

We decided to use the pulse of the trapezoidal shape, as shown in Figure 3. The pulse is symmetrical, i.e., the rise time is equal to the fall time $T_{r}=T_{f}$. The pulse delay $T_{d}$ and the voltage level *V*_1_ are set to zero.

The simplest way to perceive the filtration of control signal is to use the analysis in frequency domain. The Fourier transform of the filter response $y_{p}(t)$ to the pulse $x_{p}(t)$ is:

(12) $${\underline{Y}}_{p}\left(j\omega \right)=\underline{H}\left(j\omega \right)\cdot {\underline{X}}_{p}\left(j\omega \right),$$

where ${\underline{X}}_{p}\left(j\omega \right)$ is the Fourier transform of $x_{p}(t)$. $\underline{H}\left(j\omega \right)$ is the filter frequency response for the transfer function given by (10) when s=jω.

The amplitude spectra of the pulses is given as:

(13) $$\left|{\underline{X}}_{p}\left(j\omega \right)\right|=\frac{4U}{T_{r}\omega^{2}}\left|\sin\left(\frac{1}{2}\omega T_{r}\right)\sin\left(\frac{1}{2}\omega \left(T_{r}+T_{w}\right)\right)\right|,$$

where the zeros of this spectra are:

(14) $$\omega_{z,k}=k\frac{2\pi }{T_{r}+T_{w}}\;\mathrm{and}\;\omega_{z,m}=m\frac{2\pi }{T_{r}},\;k,\;m=1,\;2,\;\ldots$$

**Figure 4:**
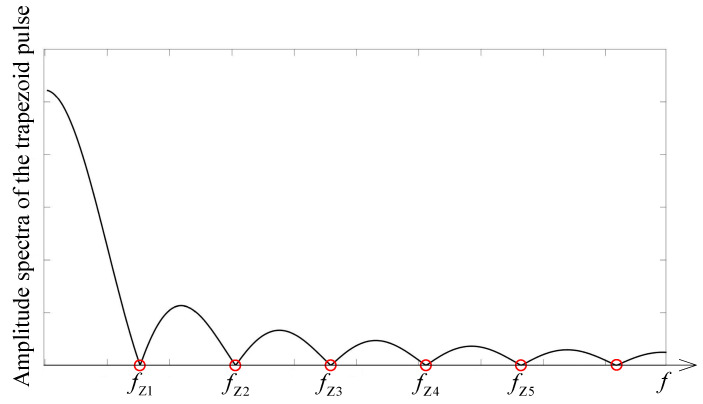
Amplitude spectra of the trapezoid pulse.

For the analyzed pulse, more than 95% of the energy spectral density is concentrated in the first five lobes of the signal amplitude spectra (see Figure 4). The fifth zero $\omega_{Z5}$ is of the form $k\frac{2\pi }{T_{r}+T_{w}}$ or $m\frac{2\pi }{T_{r}}$.

**Figure 5:**
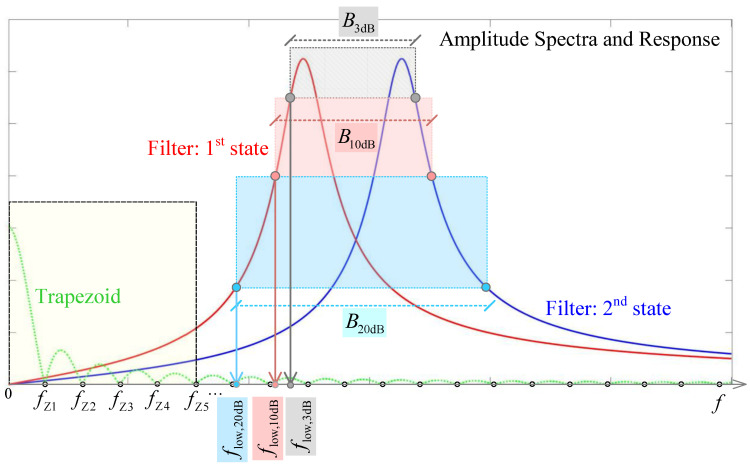
Trapezoid amplitude spectra and the filter amplitude response.

To ensure that ${\underline{Y}}_{p}\left(j\omega \right)$ could be negligible, the amplitude spectra of the pulse must be concentrated below the lower cutoff frequencies of the tunable filter. As a lower cutoff frequency, we analyzed filter bandwidths of 3 dB, 10 dB or 20 dB. We used a suppression of 20 dB where 99% of the input signal power is wasted (see Equations (8) and (9)). This means that filter has a negligible amplitude response $\left|\underline{H}\left(j\omega \right)\right|$ for $\omega \leq \omega_{low,20\;\mathrm{dB}}$. The scenario of this problem is illustrated in Figure 5, where the pulse amplitude spectra and filter amplitude response is presented. We used an assumption that $\omega_{Z5}\leq \omega_{low,20\;\mathrm{dB}}$ to be sure that the control pulse is suppressed at the filter output. This means that the minimal pulse width must be $T_{w,\min}\approx 10\pi /\omega_{low,20\mathrm{dB}}$ when $T_{r}\ll T_{w}$.

## 4. Experimental Results

Guidelines from manufacturer and experimental analysis of KnowM chips provided insights into memristor characteristics. We use self-directed-channel (SDC) memristors with tungsten (W) dopant [41], packaged in DIP-16 [33].

Before initiating the first programming cycle of a memristor, it needs to be formatted, as detailed in [15]. Small-signal memristance could be measured by applying sinusoidal voltage with an amplitude no greater than 1 V and at a frequency of 10 kHz. The memristor should be connected in series with a resistor so the voltage drop can be calculated. The resistor is also preventing the memristor from permanent damage by limiting the current. The optimal resistance value of the serial resistor is in the range from 5 to 10 kΩ.

We reviewed the available literature on memristor programming. There are different approaches to current or voltage sensing to control the memristor’s value. Also, there is an analysis of how the programming signal form and amplitude influence the memristance. The principle remains consistent: a positive or negative voltage should be applied to the resistor-memristor series to alter the memristor’s state.

Our initial step involved reproducing basic memristor programmability. We analysed simple resistor–memristor series and applied different voltage levels. Based on this experience, we continued with experiments on the circuit that we discuss in this paper. When programming the memristor from OFF to ON, we apply the *V*_2_ = 0.4 V pulse (Figure 3) at the input terminal of the circuit. For memristor programming from ON to OFF we apply the *V*_2_ = −0.4 V at the same terminal. The other pulse parameters are selected using the criteria that $f_{z5}\leq f_{low,20\;\mathrm{dB}}=9.28\;\mathrm{kHz}$, i.e., $T_{w,\min}\approx 5/f_{low,20\mathrm{dB}}\approx 0.5\;\mathrm{ms}$ for $T_{r}\ll T_{w}$, which is the minimal pulse width in order to suppress the memristor programming signal at the filter output (see Figure 5). As we used the AD2 instrumentation tool, the rise and fall times are around 2 ms, which is greater than the minimal time. The pulse parameters used are $T_{r}=T_{f}=2\;\mathrm{ms}$, $T_{w}=250\;\mathrm{ms}$, $T_{d}=0\;$, $V_{1}=0$.

For our experiments, we utilized the Analog Discovery 2 (AD2) instrumentation tool (National Instruments, Austin, TX, USA) [42]. One of the standout features of the AD2 is its ability to generate a wide range of voltage waveforms. Moreover, there is no need to manually set or configure the device, as this can be achieved using Python (ver. 3.12.0, [43]) scripts. This method ensures both flexibility and automation in signal creation. Another advantage of this tool is its capability for data collection and analysis. The AD2’s data acquisition system is robust, allowing us to capture real-time data. This was crucial for understanding the dynamic behavior of the memristor. The onboard software provided tools for data visualization and basic analysis, enabling us to make immediate interpretations. This significantly streamlined the process of studying the memristor’s behavior. However, for the final measurements, the results of which are presented here, we used the Rigol DS1054Z (RIGOL Technologies EU GmbH, Gilching, Germany) oscilloscope [44] due to its superior characteristics compared to the oscilloscope integrated within the AD2.

The choice of NE5532P as the op-amp was influenced by its low noise and distortion characteristics, making it ideal for our filter design. However, given the 5532’s sensitivity to power supply AC decoupling, it was crucial to ensure a stable power environment. To address this, we utilized the 7806 voltage regulator to ensure a stable ±6V power supply for optimal performance. Recognizing the importance of stability and noise reduction, we also incorporated appropriate decoupling capacitors both at the input and output stages of the regulator. This approach not only ensures a stable voltage output but also minimizes potential interference, optimizing the overall performance of our circuit.

**Figure 6:**
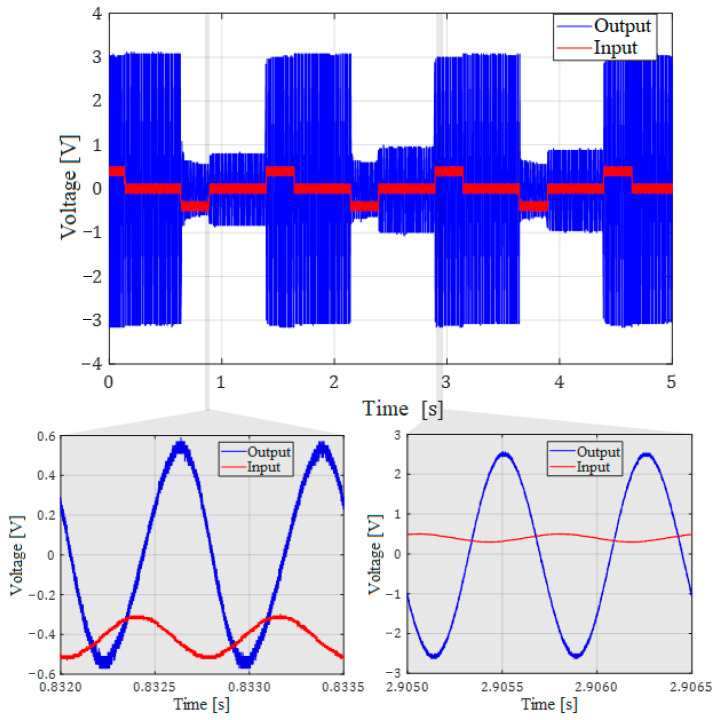
Memristor programming from 10 kΩ to 3 kΩ and vice versa during operation of the tunable active bandpass filter. Insets represent short time intervals during memristance programming.

For the experimental validation of the circuit functionality we analyzed the filter behavior during programming cycles when input signal voltages are superimposed on the memristor programming signals. The input (useful) signal was a sine wave with the amplitude of 100 mV and the frequency of 20 kHz. As an illustration in Figure 6, we present the amplitude change of the output useful signal, during the two operation states. Amplitude tuning is achieved through memristor programming to attain the desired memristance value.

**Figure 7:**
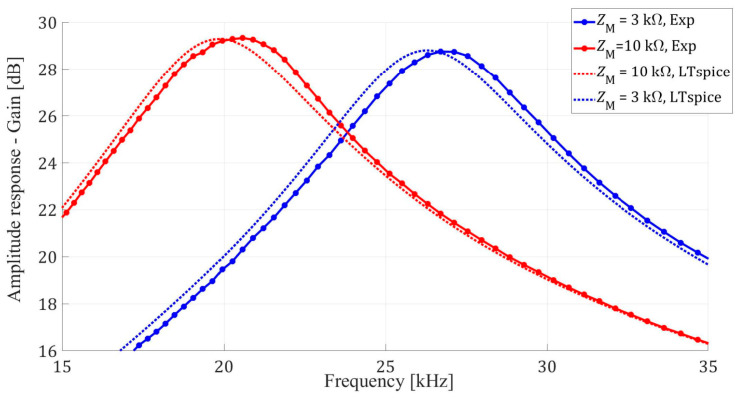
Measured amplitude response of the tunable active filter, from Figure 1, for the two memristor states.

The measured amplitude response of the filter for the two states is presented in Figure 7. The measured gains of around 29 dB at 19.5 kHz and 26 kHz are lower than the theoretical 34 dB (see Figure 2). This discrepancy can be attributed in part to parasitic shunt capacitances of 280 pF and 320 pF, respectively. These capacitances are influenced not just by the memristors with 10 kΩ and 3 kΩ memristance but also by the DIP-16 packages. This combination contributes to the observed deviation in the expected filter performance.

To further validate these findings, simulations were conducted using LTspice software (ver. 17.1.15, [45]). The LTspice models were configured to closely mimic the physical setup, including the parasitic shunt capacitances and memristance values. The simulation results were consistent with the experimental data, showing gains of approximately 29.3 dB at 19.5 kHz and 28.8 dB at 26 kHz. This adds a level of confidence to the observation that the observed differences between the theoretical and experimental gains are due to the previously mentioned parasitic elements and package influences.

By simulating the effects of parasitic capacitance and memristance in LTspice, we were able to closely match the empirical results, thereby confirming the impact of these elements on the overall filter performance.

Amplitude values of the output useful (sinusoidal) signal from Figure 6 match the corresponding amplitude values given in Figure 7.

The memristance programming signal from 10 kΩ to 3 kΩ is of the same shape as in Figure 3, but with the amplitude of *V*_2_ = 0.4 V, while for programming for from 3 kΩ to 10 kΩ, the amplitude is *V*_2_ = −0.4 V.

From the insets from Figure 6 it can be seen that the output signal does not have the programming signal component, only the useful signal.

## 5. Discussion

The advent of memristors has undeniably unveiled a plethora of opportunities in the fields of electronics, microwave engineering, artificial intelligence, and others. This breakthrough heralded a new era in electronics, where devices could potentially be more compact, more energy-efficient, and faster. However, to harness all of these improvements, a number of problems that arise when using memristors need to be resolved. Some of the main problems encountered with memristors include the variability, tunability, retention, endurance, and sensitivity to various forms of interference. Additionally, depending on the manufacturing technology, and memristors’ working principles, the programming circuits may vary. Variability here refers to the different behaviors of memristors due to different conditions during fabrication, which includes its minimal and maximal memristance values. Tunability is the ability to adjust the memristance to a precise desired value, while retention and endurance represent the memristor’s ability to maintain a specific state during longer operational time.

According to the authors’ information, KnowM is the company that produces the only commercially available memristor products. When tackling problems related to their memristors, variability cannot be influenced during the application, but improvements in the production process have been noticeable over time at KnowM. Tunability is a problem that the authors have addressed, achieving commendable results with [15] an accuracy up to 4% for low memristance values, and even better accuracy for high memristance values. In [18], it was shown that the states of KnowM memristors are stable under certain conditions and stimuli; however, the question remains as to how applicable these results are over a longer period of use.

In our previous research [18], the use of memristors in a phase shifter, amplifier, filter, and voltage divider was demonstrated. However, a separate programming circuit was used to change the states of the memristors. The advantages of circuits with memristors are primarily based on smaller parasitic inductances and capacitances of memristors compared to digital potentiometers. Nonetheless, one of the main advantages of memristors mentioned in that work was not fully utilized, which is the ability of memristors to continuously change states when applying the appropriate programming signal. All of the other switches require a small amount of idle time for the state change. Hence, the idea for the new research presented here originated: is it possible to program the memristor within an integrated circuit, without a dedicated programming circuit?

Our research explores the practical facets of incorporating memristors into active bandpass filter designs. The working principle of the filter and the idea for programming the memristors are mathematically explained. The experimental results align excellently with theoretical predictions and simulation results from a specialized tool for analyzing electrical circuits. The advantage of using memristors compared to other switching components has been demonstrated. Our findings carry significant implications for the broader field of analog systems. The ability to program a filter’s central frequency in real-time, without operational interruptions, offers a useful enhancement to adaptive systems, especially in environments where conditions change regularly.

While our results are promising, especially in the context of real-time adjustability without the need for operational pauses, it is essential to discuss the broader implications, limitations, and potential future directions of our work. Although our method is well-suited to highpass and bandpass filters, it may not be directly applicable to lowpass and notch filters. The inherent frequency characteristics of the memristor programming signal pose challenges. For lowpass filters, the programming signal, being below the filter’s cutoff frequency, would not be suppressed, leading to potential interference. Similarly, for notch filters, the programming signal remains present at the output, as it is not suppressed by the filter itself. These observations underscore the need for tailored approaches when considering different filter types.

The challenges associated with lowpass and notch filters represent a direction for continuing our future research. Exploring alternative memristor programming techniques or examining various filter architectures to mitigate interference might provide viable solutions. Furthermore, it would be interesting to explore other passbands using the same set of components as in this study but varying the memristance state, as well as utilizing different component values—or, in other words, for filters designed for alternative frequency ranges. Specifically, it would be intriguing to investigate frequency ranges that approach the maximum frequency component of the programming signal on one side, and the upper memristor operational frequency identified in our previous research on the other [18].

## 6. Conclusions

This study focuses on the pragmatic aspects of circuit design using memristors, particularly their application as tunable resistors to achieve a programmable voltage gain in an active bandpass filter. By separating the memristor programming voltage from the op-amp input, we effectively addressed the op-amp saturation issue, enabling real-time adjustability.

In this study, we first considered the circuit theoretically. It was mathematically analyzed, and its response was demonstrated through software tools. The focus was on the programming signal, which is almost entirely suppressed at the circuit output. Experimental results are in complete agreement with theoretical predictions, as shown in the paper. Deviations in the measured gain of the active filter compared to the theoretical gain are the result of the parallel parasitic capacitance of the DIP-16 package and the memristor itself. This claim has been verified by simulation in Ltspice, and the simulation result overlaps with the experimental result.

However, our experimentation did reveal some limitations, notably the frequency cap of approximately 200 kHz. This constraint is largely attributable to parasitic effects from both memristor packages and DIP-16 connectors. Improved connectors with minimized parasitics could potentially extend the operating frequencies. Additionally, the variability in memristance values remains a concern that necessitates further investigation, particularly across numerous memristors and programming cycles.

This field presents numerous opportunities for further improvement and innovation. While some promising advancements have been reported, these are not yet commercially available. Implementing a refined programming algorithm could target specific memristance values, thereby enhancing the versatility of the filter by allowing for tuning to multiple central frequencies.

The integration of memristors into electronic circuits, especially filters, is still in its nascent stages. While our research has made strides in showcasing the potential benefits, it has also highlighted areas that require further exploration. As the field progresses, we anticipate more refined solutions that can harness the full potential of memristors in a broader array of applications.

## Figures and Tables

**Table 1:** Filter specification parameters and designed memristance values.

	Specification				Designed
State	*f*_p_ [kHz]	*K*	*C* [pF]	*X*	*Z*_M_ [kΩ]
1st	19.5	50	200	150	10
2nd	26	50	200	267	3

## Data Availability

Data are contained within the article. The data presented in this study are available in this article.
